# An ex vivo Approach in European Seabass Leucocytes Supports the in vitro Regulation by Postbiotics of Aip56 Gene Expression of *Photobacterium damselae* subsp. *piscicida*

**DOI:** 10.1007/s12602-024-10255-x

**Published:** 2024-04-23

**Authors:** Marta Domínguez-Maqueda, Cristóbal Espinosa-Ruíz, María Ángeles Esteban, Francisco Javier Alarcón, Silvana T. Tapia-Paniagua, María Carmen Balebona, Miguel Ángel Moriñigo

**Affiliations:** 1 Departamento de Microbiología, Facultad de Ciencias, Instituto Andaluz de Biotecnología y Desarrollo Azul (IBYDA), Universidad de Málaga, Ceimar-Universidad de Málaga, Málaga, Spain; 2 Departamento de Biología Celular e Histología, Facultad de Biología, Universidad de Murcia, Murcia, Spain; 3 Departamento de Biología y Geología, Universidad de Almería, Ceimar-Universidad de Almería, Almería, Spain; 4 Lifebioencapsulation SL, 0413-El Alquián, Almería, Spain

**Keywords:** *Shewanella putrefaciens* Pdp11, Postbiotic, *Photobacterium damselae* subsp. *piscicida* Lg 41/01, AIP56, Cell Death, Apoptosis, Gene expression

## Abstract

*Shewanella putrefaciens* Pdp11 (SpPdp11) is a probiotic strain assayed in aquaculture; however, its postbiotic potential is unknown. Postbiotics are bacterial metabolites, including extracellular products (ECPs) that improve host physiology and immunity. Their production and composition can be affected by different factors such as the growing conditions of the probiotics. *Photobacterium damselae* subsp. *piscicida* strain Lg 41/01 (Phdp) is one of the most important pathogens in marine aquaculture. The major virulent factor of this bacterium is the exotoxin aip56, responsible for inducing apoptosis of fish leucocytes. Viable SpPdp11 cells have been reported to increase resistance to challenges with Phdp. This work aimed to evaluate the effect of two ECPs, T2348-ECP and FM1548-ECP, obtained from SpPdp11 grown under different culture conditions that previously demonstrated to exert different degradative and non-cytotoxic activities, as well as the effect on pathogens biofilm formation. These SpPdp11-ECPs were then analyzed by their effect on the viability, phagocytosis, respiratory burst and apoptogenic activity against European sea bass leucocytes infected or not with Phdp supernatant. Both ECPs, T2348-ECP and FM1548-ECP, were not cytotoxic against leucocytes and significantly reduced their apoptosis. Phagocytosis and respiratory burst of leucocytes were significantly reduced by incubation with Phdp supernatant, and not influenced by incubation with T2348-ECP or FM1548-ECP. However, both activities were significantly increased after leucocyte incubation with combined T2348-ECP and FM1548-ECP with Phdp supernatant, compared to those incubated only with Phdp supernatant. Finally, both T2348-ECP and FM1548-ECP significantly reduced the relative in vitro expression of the Phdp aip56 encoding gene.

**Supplementary Information:**

The online version contains supplementary material available at 10.1007/s12602-024-10255-x.

## Introduction

Aquaculture is a prominent sector that provides almost half of the total supply of fish products for consumption [1]. However, the intensification of aquaculture practices has increased the incidence of pathogens and diseases, and many efforts have been made to mitigate them [2, 3]. *Photobacterium damselae* subsp. *piscicida* is the causative agent of photobacteriosis or pasteurellosis, one of the most important diseases affecting a wide range of marine fish species worldwide [4, 5], such as gilthead seabream (*Sparus aurata*), European seabass (*Dicentrarchus labrax*) and Senegalese sole (*Solea senegalensis*) [6–8]. *P. damselae* subsp. *piscicida*, including the strain of this study *Photobacterium damselae* subsp. *piscicida* Lg41/01 (Phdp), is able to invade non-phagocytic cells and evade the immune response [9]. This pathogen also possesses virulence factors for host iron acquisition [10–12] and is capable of modulating host complement activity [13] and superoxide radical production [14]. In addition, all virulent strains contain the plasmid carrying the *aip56* toxin gene [15], which encodes the AIP56 exotoxin thought to be responsible for inducing apoptosis of fish macrophages and neutrophils [11, 16]. To fight to this, and other fish diseases, antimicrobials are used in food animals and aquaculture, and their use can be categorized as therapeutic against bacterial infections. However, the use of antimicrobials in aquaculture may involve a broad environmental application that affects a wide variety of bacteria, promoting the spread of bacterial resistance genes [17]. To this end, the development of alternative strategies against fish bacterial diseases, including probiotics, are essential to achieve a sustainable and environmentally friendly aquaculture industry [18, 19].

Probiotics are live microorganisms that confer a health benefit to the host when administered in adequate amounts [3]. Such is the case of *S. putrefaciens* Pdp11 (SpPdp11), a strain isolated from skin of healthy gilthead seabream (*Sparus aurata*, L.), which has been proposed as a probiotic to induce beneficial effects when dietary administered to farmed gilthead seabream and Senegalese sole (*Solea senegalensis*, Kaup) [reviewed by [20, 21]. Regarding its effects on pathogens and diseased animals, the probiotic SpPdp11 has been shown to increase resistance against Phdp [9, 22]. In addition, SpPdp11 showed the ability to reduce Phdp in vitro adhesion to the skin and intestinal mucosa of Senegalese sole [23].

As above, while SpPdp11 viable cells as a probiotic showed promising results, their postbiotic potential is still unknown. Postbiotics have been shown to mimic the health benefits of probiotics, resulting in a safer and more stable alternative compared to products that require live microorganisms to be functional [24]. The International Scientific Association for Probiotics and Prebiotics (ISAPP) convened a panel that defined postbiotics as a “preparation of inanimate microorganisms and/or their components that confers a health benefit to the host” [25]. Therefore, postbiotics represent a great alternative option when it comes to biological approaches to disease control [26].

In addition, postbiotics can be influenced by different factors, such as the culture conditions where they are obtained, allowing them to be optimized and applied for different purposes and objectives [27]. This is in accordance with owned-recent studies where SpPdp11 was growing under different cultivation conditions, and their extracellular products (ECPs; SpPdp11-ECPs) were obtained as potential postbiotics that were subjected to different trials. Therefore, among all these SpPdp11-ECPs, two conditions, T2348-ECP and FM1548-ECP, were selected for the present study. T2348-ECP were ECPs obtained when SpPdp11 was culture on tryptone soja agar medium supplemented with NaCl (TSAs; T media) after incubation at 23 ºC for 48 h. Additionally, FM1548-ECP were ECPs obtained when SpPdp11 was culture on a media consisted of a partial replacement of aquafeed by 25% of a blend of microalgae (*Chlorella fusca, Tisochrysis galbana, Microchloropsis gaditana* and *Arthrospira platensis*) (FM media), after incubation at 15 ºC for 48 h. Both ECP conditions, T2348-ECP and FM1548-ECP, were selected because of exhibiting the best in vitro capabilities regarding important degradative and non-cytotoxic activities, as well as effects on pathogen biofilm formation [28] and quorum-quenching capacity. These abilities could be related with the production of destructive and/or disrupted enzymes interfering in the infection process [29–31] of Phdp.

In this way, the present work considered both SpPdp11-ECPs, T2348-ECP and FM1548-ECP, for evaluating their effect on the Phdp virulence. For this, an ex vivo approach was firstly evaluated regarding the effect of the aforementioned SpPdp11-ECPs on the viability, respiratory burst, phagocytic activity and apoptosis undergo by European sea bass head kidney leucocytes (HKLs) challenged with Phdp supernatant (Phdp-ECPs). In addition, since the pathogenicity of Phdp is closely related to its exotoxin AIP56, the effect of T2348-ECP and FM1548-ECP on the relative in vitro transcription of the *aip56* gene was also analysed. These findings will emphasize SpPdp11 posbiotics’ effect and potential current role on European seabass aquaculture and provide key findings to promote future research.

## Materials and Methods

### Bacterial Strains, Media and Culture Conditions

*S. putrefaciens* Pdp11 (SpPdp11) CECT 7627 was selected based on its in vitro and in vivo ability to exert beneficial effects as a probiotic on gilthead seabream and Senegalese sole specimens. SpPdp11 was cultured on tryptic soy agar supplemented with NaCl (1.5%) (TSAs) at 23 ºC for 24 h. One or two colonies were then transferred to 100 mL flasks containing 50 mL of tryptic soy broth (Oxoid Ltd Basingstoke, UK) supplemented with NaCl (1.5%) (TSBs) and incubated at 23 ºC, 36 h, on shaking (2 x g) (10^9^ UFC/mL, onset of stationary phase).

*P. damselae* subsp. *piscicida* strain Lg 41/01 (Phdp) was isolated from diseased cultured Senegalese sole [32] and cultured on TSAs plates at 23 ºC for 48 h. Then, one or two colonies were transferred to TSBs 10 mL tubes and incubated at 23 ºC for 18 h, on shaking (2 x g) until mid-exponential phase (OD_600nm_ = 0.8 ~ 10^4^ UFC/mL) [11].

### Extracellular Product Extraction from SpPdp11 (SpPdp11-ECPs) Grown under Different Culture Conditions

**Fig. 1:**
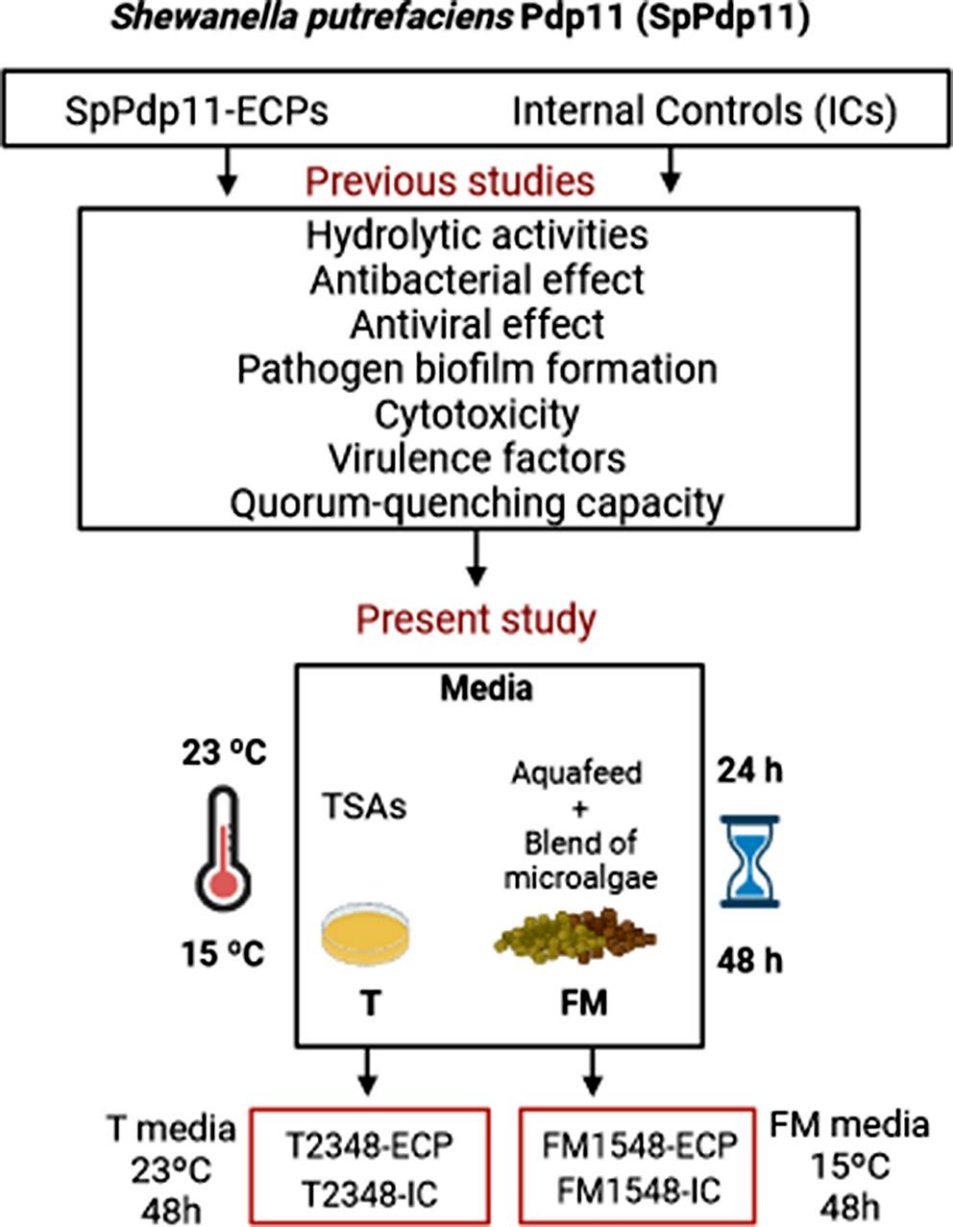
Different conditions for ECP extraction and nomenclature used in this experiment. Different SpPdp11-ECPs conditions were previously analysed by their: hydrolytic activities, antibacterial and antiviral effects, effect on pathogen biofilm formation, cytotoxicity against different fish cell lines, virulence factors and quorum-quenching capacities. The SpPdp11-ECPs with the best results obtained before, were used for the present study, specifically two SpPdp11-ECP conditions, inside the red box: T2348-ECP and FM1548-ECP, and their respective internal controls, that are named equally but adding “Internal control (IC)” (T2348-IC and FM1548-IC). Letters in the nomenclature indicate culture medium (T or FM) and numbers stand for temperature (ºC) and incubation time (h).

Extracellular products (ECPs) from solid medium cultures were obtained by the cellophane plate technique [33]. In brief, volumes (1 mL) of SpPdp11 cultures described in Sect. 2.1 were spread over sterile cellophane sheets placed on TSAs plates (T media). Similarly, another 1 mL volume was spread on sterile cellophane sheets placed on plates containing a partial replacement of aquafeed by 25% of a blend of microalgae (*Chlorella fusca, Tisochrysis galbana, Microchloropsis gaditana* and *Arthrospira platensis*) (160 g/L) and agar (1.5% w/v) (FM media). The experimental aquafeeds were elaborated by the Experimental Diets Service (CEIMAR, University of Almeria, Spain) using a two-screw extruder (Evolum 25, Clextral, France) (Table S1). Aquafeed used was formulated for mimicking commercial diets, and the supplemented-microalgae diet included 25% of the above-mentioned blend of microalgae. Aquafeed was incorporated and used for probiotic growth in order to approach ECP production by the probiotics when grown on the feed of farmed fish. The partial replacement of a blend of microalgae were included as an alternative and environmentally sustainable source of feed ingredients in aquaculture [34]. The objective of assessing the effects of SpPdp11 temperature and incubation time on ECPs secretion was addressed by incubating all inoculated plates at 15 ºC and 23 ºC (culture temperature range for gilthead seabream [35] and Senegalese sole [36]), and for 24–48 h (early and late cultures, respectively). All assayed media, but without SpPdp11 inoculation, were incubated under the same temperature and time conditions described above and used as internal controls (ICs) to check for potential media background. Assayed conditions are summarized in Fig. 1.

Bacterial cells from the different culture conditions and ICs were harvested after 24 h and 48 h incubation with 2 mL sterile phosphate-buffered saline (PBS, pH 7.2), centrifuged (10,000 x g, 20 min, 4 ºC) and the supernatants were filtered through 0.45 and 0.2 μm pore-size membrane filters (Merck Millipore, USA) to obtain ECPs. Controls were harvested similarly, but from non-inoculated media. ECPs were also concentrated using Amicon Ultra centrifugal filters (10 K) (Merck Millipore, USA). Protein concentration was determined using Qubit Protein assay kits and the Qubit 2.0 (Thermo Fisher Scientific, USA). Absence of microbial growth was checked on TSAs plates inoculated with ECP aliquots and incubated for 24–48 h at 23 ºC. ECPs were stored at -80 ºC until use.

### Effects of SpPdp11-ECPs on Phdp Growth

Minimum Inhibitory Concentration (MIC) of SpPdp11-ECPs, T2348-ECP and FM1548-ECP, against Phdp was assayed to determine the potential inhibition of Phdp bacterial growth. For this, Phdp was grown on TSAs plates at 23 ºC for 48 h. Bacterial cells were collected and suspended in 10 mL TSBs tubes to achieve an OD 595 nm ~ 0.5. Then, 20 µL of bacterial suspensions were pipetted into flat-bottom polystyrene 96-well plates (#D51588, Sarstedt, Nümbrecht, Germany), filled up to 200 µL with TSBs and used as positive control (Phdp Control +). Simultaneously, to determine the MIC of T2348-ECP and FM1548-ECP, 20 µL of Phdp bacterial suspensions were pipetted, and microplate wells filled up to 200 µL final volume by adding 90 µL of TSBs double concentrated and 90 µL of ten-fold dilutions of T2348-ECP and FM1548-ECP, separately (initial protein content adjusted to 30 µg/mL) (Phdp + T2348-ECP and Phdp + FM1548-ECP). Both ECPs were added at the beginning (0 h) of incubation and growth was determined after 48 h incubation by absorbance values (OD595 nm) in a plate reader (Multiskan FC, Thermo Fisher). The same protocol was carried out to test ICs of each ECP condition (Phdp + T2348-IC and Phdp + FM1548-IC). Each value was subtracted from the corresponding control cell values, containing only the culture medium. We conducted three independent experiments, with five technical replicates (*n* = 5 wells) per condition in each assay.

### Extracellular Products Extraction from Phdp (Phdp-ECPs) Grown in Presence of SpPdp11-ECPs

**Fig. 2:**
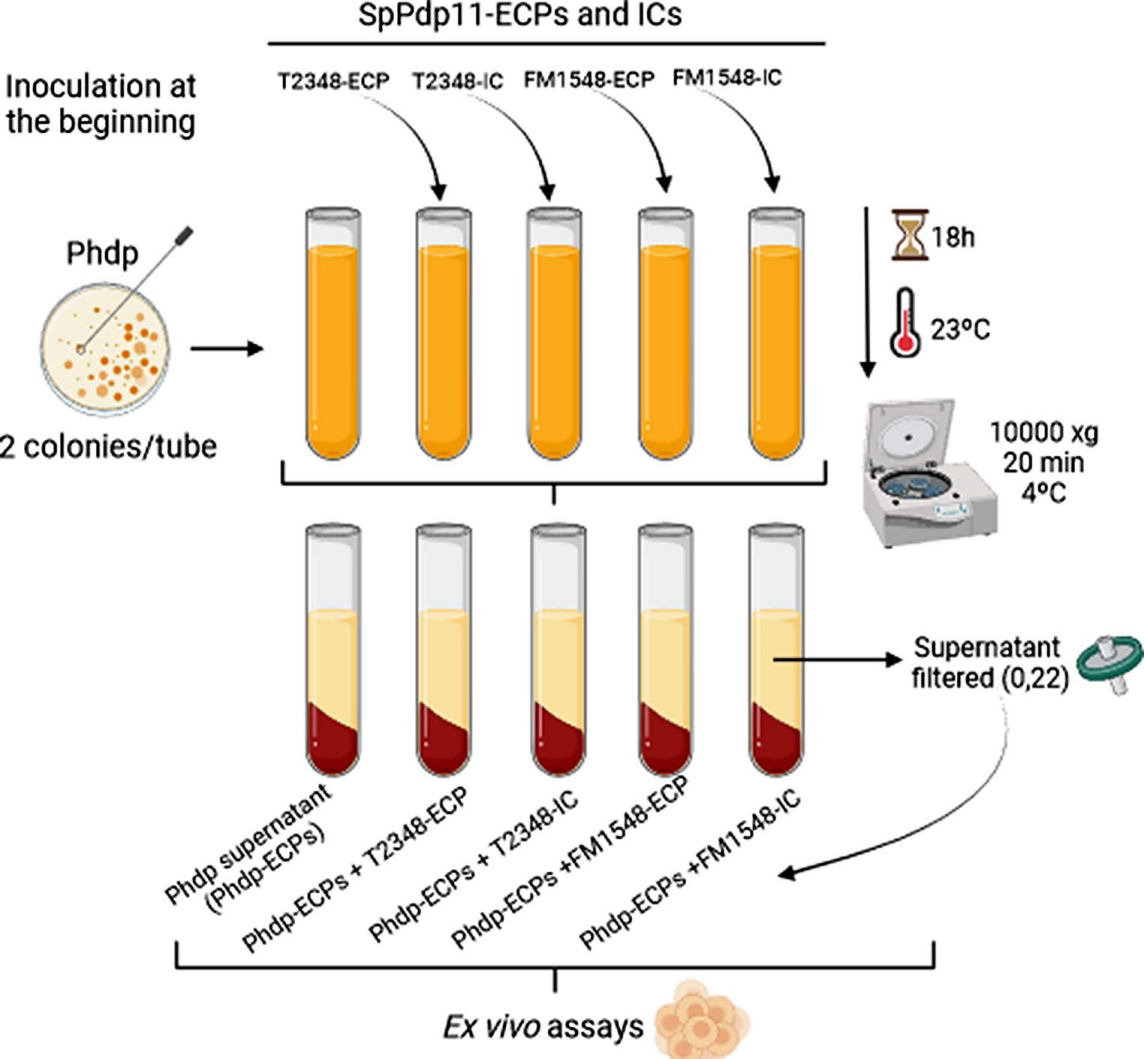
Protocol of extraction of Phdp supernatant alone (Phdp-ECPs) and after adding SpPdp11-ECPs (T2348-ECP and FM1548-ECP) and ICs (T2348-IC and FM1548-IC)

As explained above, Phdp was grown on TSAs plates at 23 ºC for 48 h. Then, two Phdp colonies were inoculated to 10 mL tubes of TSBs. Dilutions of T2348-ECP and FM1548-ECP that did not affect Phdp growth were selected (N dilutions). Then, Phdp was grown in the presence of both, T2348-ECP and FM1548-ECP, which were added at the beginning (0 h) of Phdp incubation at 23 ºC for 18 h under agitation (120 x g). Finally, the supernatant of Phdp (Phdp-ECPs) was obtained as a combination; Phdp-ECPs + T2348-ECP and Phdp-ECPs + FM1548-ECP. Simultaneously, 10 mL tubes of Phdp cultures were also added ICs of the selected SpPdp11-ECPs and were incubated equally (Phdp-ECPs + T2348-IC and Phdp-ECPs + FM1548-IC). A Phdp culture without ECPs nor ICs was maintained as a positive control (Phdp-ECPs) (Fig. 2). Then, bacterial cells from the different culture conditions were centrifuged (10,000 xg, 20 min, 4 ºC) and the supernatants were filtered through 0.2 μm pore-size membrane filters (Merck Millipore, USA) to obtain the Phdp supernatant alone (Phdp-ECPs) or added with ECPs (Phdp-ECPs + T2348-ECP and Phdp-ECPs + FM1548-ECP) or ICs (Phdp-ECPs + T2348-IC and Phdp + FM1548-IC). This Phdp-ECP extraction was necessary to obtain the apoptosis-inducing protein AIP56, since it is an extracellular secreted AB-type toxin [16]. Protein concentration of the supernatant was determined using Qubit Protein assay kits and the Qubit 2.0 (Thermo Fisher Scientific, USA). To ensure the absence of growth, aliquots of the different ECP samples were cultured on TSAs plates and incubated at 23 ºC for 24–48 h. All ECPs were stored at -80 ºC until use.

### Head Kidney Leucocyte Isolation and FDA/PI Flow Cytometry Assay

Twelve European sea bass specimens (106.3 ± 27.0 g mean body weight) were obtained from a local farm (Murcia, Spain) and maintained into two running seawater aquaria (*n* = 6) (250 L, flow rate 900 L/h), at the Marine Fish Facilities, University of Murcia (Spain). The protocols were authorized by the Ethical Committee of the University of Murcia (protocol code A13150104) following the regulations of European Union for animal handling (2010/63/EU). Water parameters were maintained at 28‰ salinity and 20 ºC temperature, and with an artificial photoperiod (12 L:12D). Fish were quarantined for four weeks and fed the commercial feed (Skretting, Burgos, Spain) at a rate of 1.5% body weight/day. Specimens were killed by an overdose of anaesthetic (MS222, 100 mg/mL; Sandoz), exsanguinated and head-kidney (HK) samples were obtained. HK leucocytes (HKLs) were obtained [37]. Briefly, HK fragments were passed through a nylon mesh (mesh size 100 μm pore size) using 12 mL of L-15 [Leibovitz culture medium (Gibco) supplemented with 10% foetal calf serum (FCS, Gibco), 100 i.u./mL penicillin (Flow) and 100 mg/mL streptomycin (Flow)]. The HKLs were then washed twice (400 x *g*, 10 min, room temperature), counted (Automated Cell Counter TC20, Bio-Rad) and adjusted to 10^7^ cells/mL in L-15. Cell viability was determined by trypan blue exclusion test. Aliquots of 100 µL of HKL suspensions were incubated in a plate with: Phdp-ECPs, SpPdp11-ECPs (T2348-ECP and FM1548-ECP) and their ICs (T2348-IC and FM1548-IC), and combinations of Phdp-ECPs and SpPdp11-ECPs (Phdp-ECPs + T2348-ECP and Phdp-ECPs + FM1548-ECP) or ICs (Phdp-ECPs- + T2348-IC and Phdp-ECPs + FM1548-IC) (protein content adjusted to 30 µg/mL) at 25 ºC, 24 h, 120 r.p.m. After incubation, the samples of HLKs were transferred to cytometer tubes. Viable and non-viable cells were identified by simultaneous assessment of propidium iodide (PI) and fluorescein diacetate (FDA) fluorescence [38]. After 24 h, 10 µL of FDA (0.5 µg/mL) were added to each tube. The samples were incubated at room temperature in the dark for 30 min, and then 10 µL of PI in isotonic saline was added to each tube to a final concentration of 50 µg/mL. The tubes were then immediately placed on ice and kept refrigerated during flow cytometric analysis. These cells were identified by observing green (FDA), red (PI) or both (FDA-PI) intracellular fluorescence in a flow cytometer (Becton Dickinson) with an argon-ion laser adjusted to 488 nm. The analyses were performed on 10,000 cells, which were acquired at a rate of 300 cells/s. The quantitative study of the flow cytometric results was made using the statistical option of the Lysis Software Package (Becton Dickinson). All the analyses were performed in triplicate.

### Cellular Immune Parameters

#### Phagocytic Activity

Phagocytosis of *Saccharomyces cerevisiae* cells (strain S288C) by HKLs was studied by flow cytometry according to Rodriguez et al. [39]. Heat-killed and lyophilized yeast cells were labelled with fluorescein isothiocyanate (FITC, Sigma), washed, and adjusted to 10^8^ yeast cells/mL in L-15 medium. Phagocytosis samples consisted of 60 µL labelled-yeast cells and 100 µL of HKLs previously incubated with Phdp-ECPs, SpPdp11-ECPs (T2348-ECP and FM1548-ECP) and their ICs (T2348-IC and FM1548-IC), and combinations of Phdp-ECPs and SpPdp11-ECPs (Phdp-ECPs + T2348-ECP and Phdp-ECPs + FM1548-ECP) or ICs (Phdp-ECPs + T2348-IC and Phdp-ECPs + FM1548-IC) in L-15. Samples were mixed, centrifuged (22 ºC, 5 min, 400 x *g*), resuspended and incubated (22 °C, 30 min). Afterwards, samples were placed on ice to stop phagocytosis and 400 mL ice-cold PBS was added to each sample. The fluorescence of the extracellular yeasts was quenched by adding 50 µL ice-cold trypan blue (0.5% in PBS). Standard samples of FITC-labelled *S. cerevisiae* or HKLs were included in each phagocytosis assay. All samples were analysed in a flow cytometer. Analyses were performed on 3000 cells and the data collected in the form of two-parameter side scatter (granularity) (SSC), forward scatter (size) (FSC) and green fluorescence (FL1). Dot plots, or histograms were made on a computerised system. The fluorescence histograms represented the relative fluorescence on a logarithmic scale. The cytometer was set to analyse the phagocytic cells, showing highest SSC and FSC values. Phagocytic ability was defined as the percentage of cells with one or more ingested bacteria (green-FITC fluorescent cells) within the phagocytic cell population, whilst the phagocytic capacity was the mean fluorescence intensity. The quantitative study of the flow cytometry results was made using the statistical option of the Lysis Software Package.

#### Respiratory Burst Activity

Effects of selected ECPs on HKL respiratory burst activity was studied by using a chemiluminescence method [40]. Briefly, 100 µL of HKL suspension were placed in triplicate in wells of a 96-well flat-bottomed plate. Then, 100 µL of HBSS containing 1 mg/mL phorbol myristate acetate (PMA, Sigma) and 10^− 4^ M luminol (Sigma-Aldrich) were added to each well. The plate was shaken and immediately read in a plate reader for 1 h at 2 min intervals. The kinetic of the reactions was analysed and the maximum slope of each curve was calculated. Luminescence backgrounds were calculated using reactant solutions containing luminol but not PMA.

### Effects of SpPdp11-ECPs on the Relative Phdp in Vitro aip56 Gene Expression

Phdp Lg 41/01 was grown on TSAs plates at 23 ºC for 48 h. Then, one or two Phdp colonies were inoculated to 10 mL tubes of TSBs. To determine the effect of SpPdp11-ECPs (T2348-ECP and FM1548-ECP) and their ICs (T2348-IC and FM1548-IC) on the relative Phdp in vitro aip56 gene expression, dilutions of T2348-ECP and FM1548-ECP (N dilutions) that did not inhibit Phdp bacterial growth were added at the beginning (0 h) of incubation of these 10 mL tubes, that were incubated at 23 ºC for 18 h under agitation (120 x g). Simultaneously, 10 mL tubes of Phdp cultures were also added ICs, T2348-IC and FM1548-IC, and were incubated equally. A Phdp culture without SpPdp11-ECPs nor ICs was maintained as a positive control. For each culture, the cells were harvested after 18 h of incubation by centrifugation at 5000 x g for 10 min at 4 ˚C. Three independent experiments were carried out with five technical replicates (*n* = 5 tubes).

Then, RNA was extracted from the bacterial cells with the RNA Purification Kit (#K0731 ThermoScientific™, Madrid, Spain) according to the manufacturer’s instructions. The quality of the RNA was determined by using 2X RNA Loading Dye kit (#R0641 ThermoScientific™), and 2X loading buffer added in a 1:1 ratio to 2 µL of previously extracted RNA. The mixture was subjected to heat shock at 95 °C for 5 min. RNA quality was checked by running an aliquot on agarose gels (1% w/v). Subsequently, extracted RNA was quantified with the Qubit 2.0 High Sensitivity quantification kit (Thermo Scientific, Madrid, Spain). RNA was stored at -80 ºC until use. Finally, cDNA was obtained from 100 ng RNA of each sample by using the Maxima First Strand cDNA Synthesis Kit for RT-qPCR with dsDNase and random primers (#K1671 Thermo Scientific) according to manufacturers’ instructions. cDNA obtained was also stored at -20 °C until use.

Relative transcription of the gene encoding AIP56 toxin was determined by using qRT-PCR and 16 S rRNA was used as a reference gene for performing relative quantification as described Nuñez-Díaz et al. [11]. RT-qPCR reactions were performed in a CFX96 Touch Real-Time PCR Detection System (Bio-Rad Laboratories, Hercules, CA, USA). Reaction mixture contained 2 µl of cDNA, 50 U of Taq Accustart II Trough Mix (Boimerieux, Marcy-l’Étoile, France), 20 pmol of *aip56*_R primer 5′- CGGCAGTGAATTAGGCTTTCT-3′ and 20 pmol of *aip56*_F primer 5′- CCGCCTCCGTTGAAATCATCC-3′ in 20 µL final volume. The primers used for the genes assayed in this work were obtained from Nuñez-Diaz et al. [11]. The PCR program consisted in initial denaturation cycle at 95 ºC for 60 s, followed by 40 cycles at 95 ºC for 30 s, 55 ºC for 40 s and 72 ºC for 60 s. Amplification was followed by a standard melting curve from 65 ºC to 95 ºC, in increments of 0.5 ºC for 5 s at each step, to confirm that only one product was amplified and detected. Samples were run in parallel with 16 S rRNA reference gene. The change in gene expression under the different growth conditions was recorded as comparative Cq (2^−ΔΔCt^) [41] normalized to the reference gene and relative to Phdp cells grown in TSBs.

### Statistical Analysis

Statistical analyses were conducted using IBM SPSS Statistics 22.0. Normality and homogeneity of variance of the data were determined by using Shapiro-Wilk and Levene’s tests, respectively. Differences were statistically analyzed by one-way analysis of variance (ANOVA) with Tukey and Games-Howell *post hoc* tests when statistical requirements were fulfilled. Non-normally distributed data were analyzed with the non-parametric Kruskal-Wallis test, followed by a multiple comparison test. Statistical significance was set for *p* ≤ 0.05.

## Results

### Phdp Growth and Minimum Inhibitory Concentration (MIC)

**Fig. 3:**
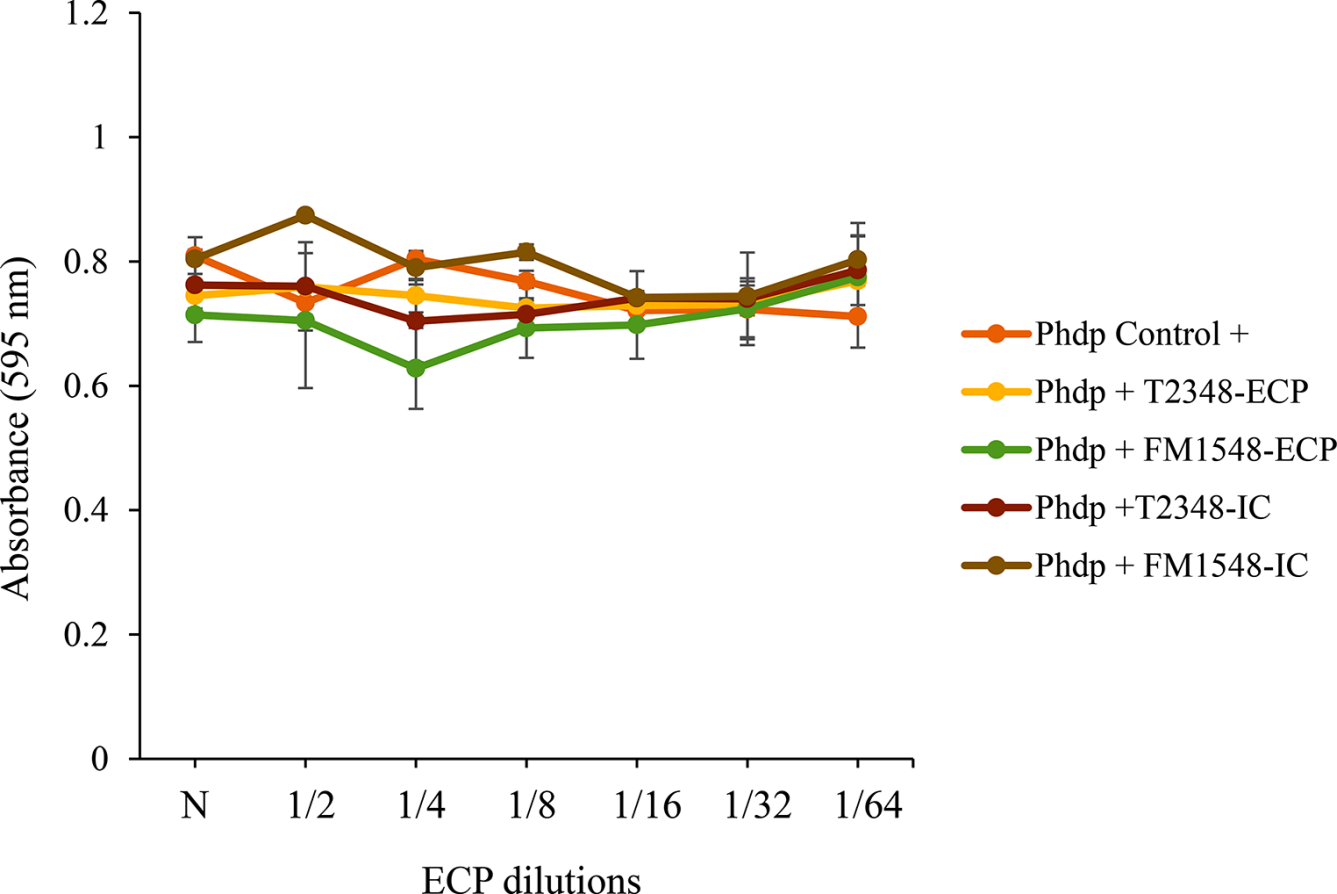
Growth of Phdp (absorbance values, OD 595 nm) after 48 h incubation with ECP serial dilutions (µg protein µL-1) extracted from *S. putrefaciens* Pdp11 probiotic strain cultured under different conditions. “Phdp Control +” indicates growth without ECPs nor ICs added, and “Phdp +” indicates growth with the different SpPdp11-ECPs (Phdp + T2348-ECP and Phdp + FM1548-ECP) and ICs (Phdp + T2348-IC and Phdp + FM1548-IC) added. Letters in the nomenclature indicate the culture medium (T: TSAs; FM: mix of aquafeed and a blend of microalgae added medium) and numbers stand for temperature (ºC) and incubation time (h). The results are representative of three independent experiments and are expressed as mean ± SD (*n* = 5). N: undiluted ECPs or ICs

None of the SpPdp11-ECPs obtained after growth of the probiotic strain SpPdp11 on the different media and culture conditions assayed inhibited Phdp cell growth (Fig. 3). ICs neither inhibited the Phdp growth. For this reason, undiluted SpPdp11-ECPs and ICs (N dilutions) were used for all subsequent experiments.

### HKLs Cell Death

**Fig. 4:**
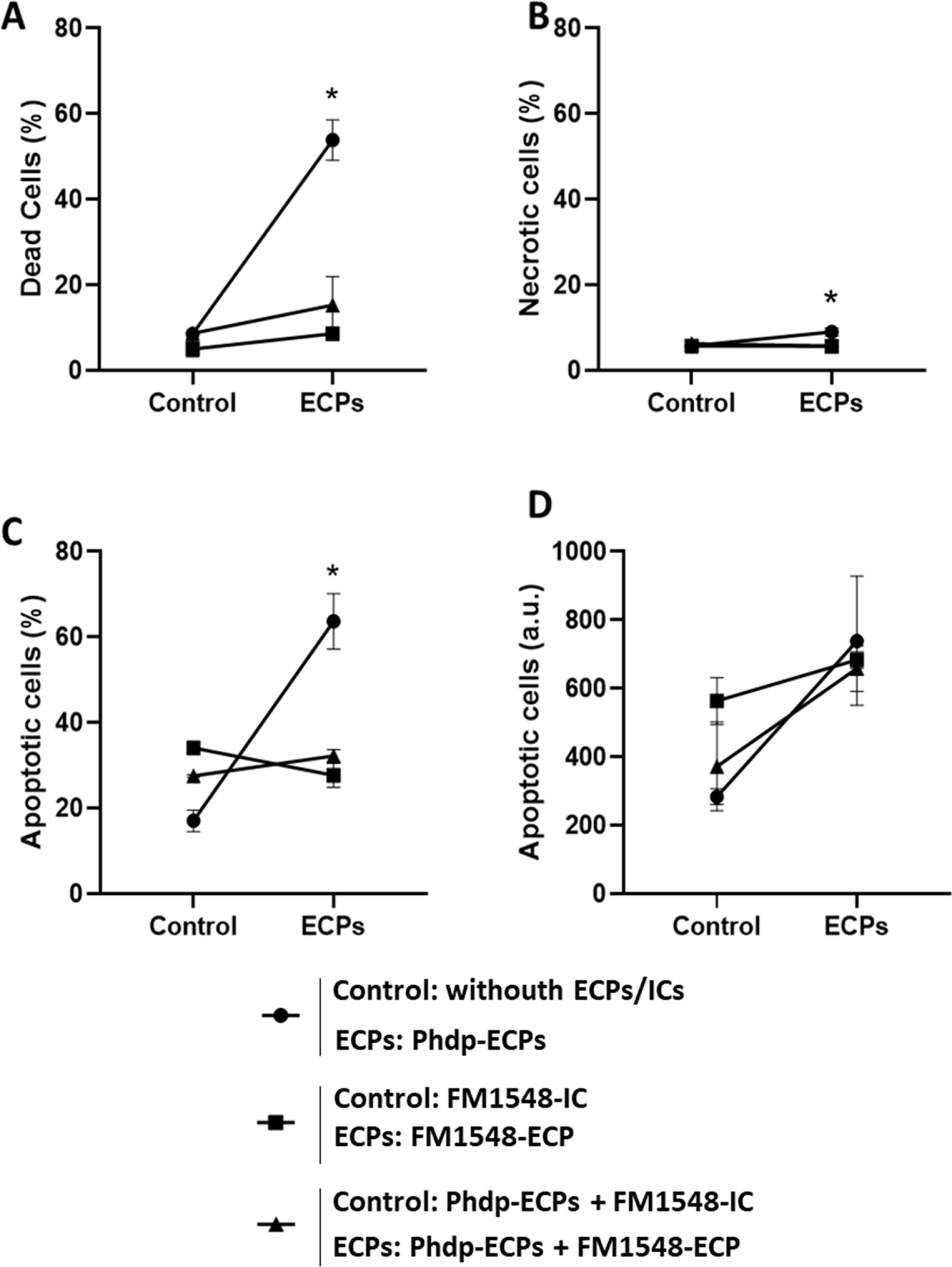
Cell death of European sea bass head kidney leucocytes (HKLs) after 24 h incubation with SpPdp11-ECPs and its respective ICs. Circles indicate: Control (HKLs without ECPs nor ICs) and ECPs (HKLs + Phdp-ECPs); Squares indicate: Control (HKLs + FM1548-IC) and ECPs (HKLs + FM1548-ECP); Triangles indicate: Control (HKLs + Phdp-ECPs + FM1548-IC) and ECPs (HKLs + Phdp-ECPs + FM1548-ECP). **A**. Percentage of FDA+/PI + + FDA-/PI+ (dead cells), **B**. Percentage of FDA-/PI + (necrotic cells), **C**. Percentage of FDA+/PI- (apoptotic cells) and **D**. Intensity of FDA+/PI- (apoptotic cells) (arbitrary units. a.u.). Results are expressed as mean ± SEM (*n* = 3; 10,000 events). Asterisks (*) mean statistically significant differences (*p* < 0.05) between groups (Control and ECPs)

When HKLs were incubated with the supernatant of Phdp (Phdp-ECPs) for 24 h, the presence of dead cells was observed (approximately 58%) (Fig. 4A). However, when HKLs were exposed to FM1548-ECP or the combine Phdp-ECPs + FM1548-ECP, no mortality was observed, and the percentage of dead cells was significantly lower compared to HKLs incubated with Phdp-ECPs (Fig. 4A).

The percentage of necrotic cells was always below 10% (Fig. 4B). This percentage was significantly lower after the incubation of HKLs with FM1548-ECP and the combine Phdp-ECPs + FM1548-ECP, compared to the values observed when HKLs were exposed to the Phdp supernatant, Phdp-ECPs (Fig. 4B). Regarding apoptotic cells, HKLs incubation with Phdp-ECPs resulted in high percentage of apoptosis (more than 60%) it being significantly higher compared to values of apoptotic cells obtained when HKLs samples were incubated with FM1548-ECP alone or with the supernatant of Phdp (Phdp-ECPs + FM1548-ECP) (Fig. 4C). However, no significant differences were observed in HKL samples incubated with Phdp-ECPs, FM1548-ECP or FM1548 + Phdp-ECPs when apoptosis was considered as arbitrary units (a.u.) (Fig. 4D).

**Fig. 5:**
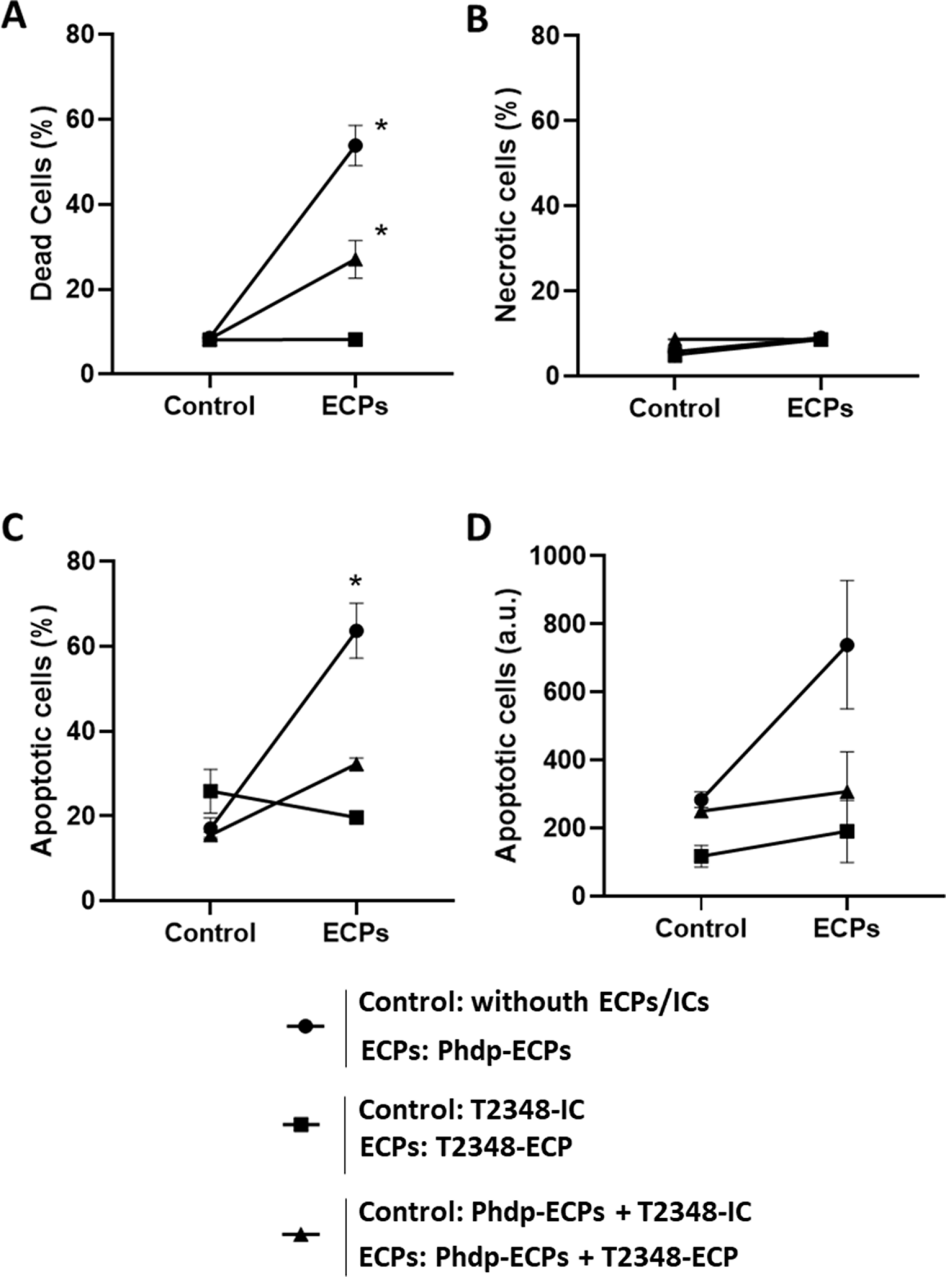
Cell viability parameters of European sea bass head kidney leucocytes (HKLs) after 24 h incubation with SpPdp11-ECPs and its respective ICs. Circles indicate: Control (HKLs without ECPs nor ICs) and ECPs (HKLs + Phdp-ECPs); Squares indicate: Control (HKLs + T2348-IC) and ECPs (HKLs + T2348-ECP); Triangles indicate: Control (HKLs + Phdp-ECPs + T2348-IC) and ECPs (HKLs + Phdp-ECPs + T2348-ECP). **A**. Percentage of FDA+/PI + + FDA-/PI+ (dead cells), **B**. Percentage of FDA-/PI + (necrotic cells), **C**. Percentage of FDA+/PI- (apoptotic cells) and **D**. Intensity of FDA+/PI- (apoptotic cells) (arbitrary units. a.u.). Results are expressed as mean ± SEM (*n* = 3; 10,000 events). Asterisks (*) mean statistically significant differences (*p* < 0.05) between groups (Control and ECPs)

Similar results were observed in assays performed with T2348-ECP both, alone or combined with the supernatant of Phdp (T2348-ECP + Phdp-ECPs) (Fig. 5). The highest percentage of dead cells was obtained when HKLs were incubated with the supernatant of Phdp (Phdp-ECPs) whereas the lowest values were observed when HKLs were exposed to T2348-ECP. No significant differences regarding cell death were observed between T2348-ECP and the combined Phdp-ECPs + T2348-ECP after HKLs incubation (Fig. 5A). However, significant differences were observed when Phdp supernatant was combined with T2348-ECP (Phdp-ECPs + T2348-ECP) or T2348-IC (Phdp-ECPs + T2348-IC) (Fig. 5A). The percentage of necrotic cells was always low and no significant differences were observed between treatments (Fig. 5B). The percentage of apoptotic HKLs significantly increased after incubation with Phdp supernatant (Phdp-ECPs), compared to those incubated with T2348-ECP alone or combined with Phdp supernatant (Phdp-ECPs + T2348-ECP) (Fig. 5C). However, no significant differences were observed in the apoptosis of the samples, when studied as a.u. although the highest values were obtained for HKLs incubated with Phdp supernatant (Phdp-ECPs) (Fig. 5D).

### Cell-Mediated Immunity: Phagocytosis and Respiratory Burst

**Fig. 6:**
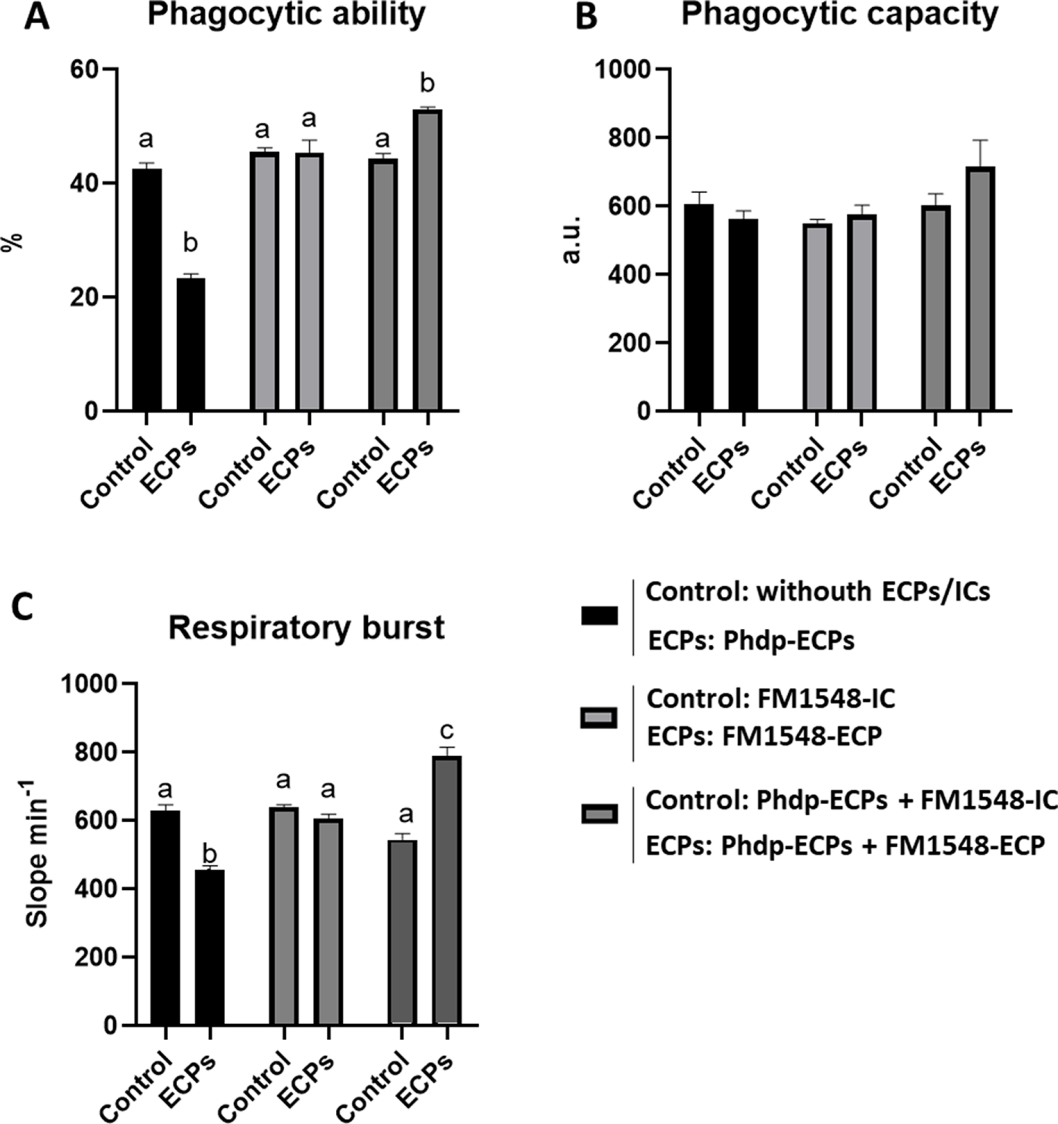
Cellular innate immunity parameters of European sea bass (*Dicentrarchus labrax*) head kidney leucocytes (HKLs) incubated for 24 h with different ECPs and ICs. Bars from left to right indicate: Control (HKLs) and ECPs (HKLs + Phdp-ECPs); Control (HKLs + FM1548-IC) and ECPs (HKLs + FM1548-ECP); Control (HKLs + Phdp-ECPs + FM1548-IC) and ECPs (HKLs + Phdp-ECPs + FM1548-ECP). **A**. Phagocytic ability, **B**. Phagocytic capacity and **C**. Respiratory burst. Results are expressed as means and error bars in the columns indicate the standard error of the means (*n* = 6). Different letters indicate significant differences (*p* < 0.05) between groups (Control and ECPs)

**Fig. 7:**
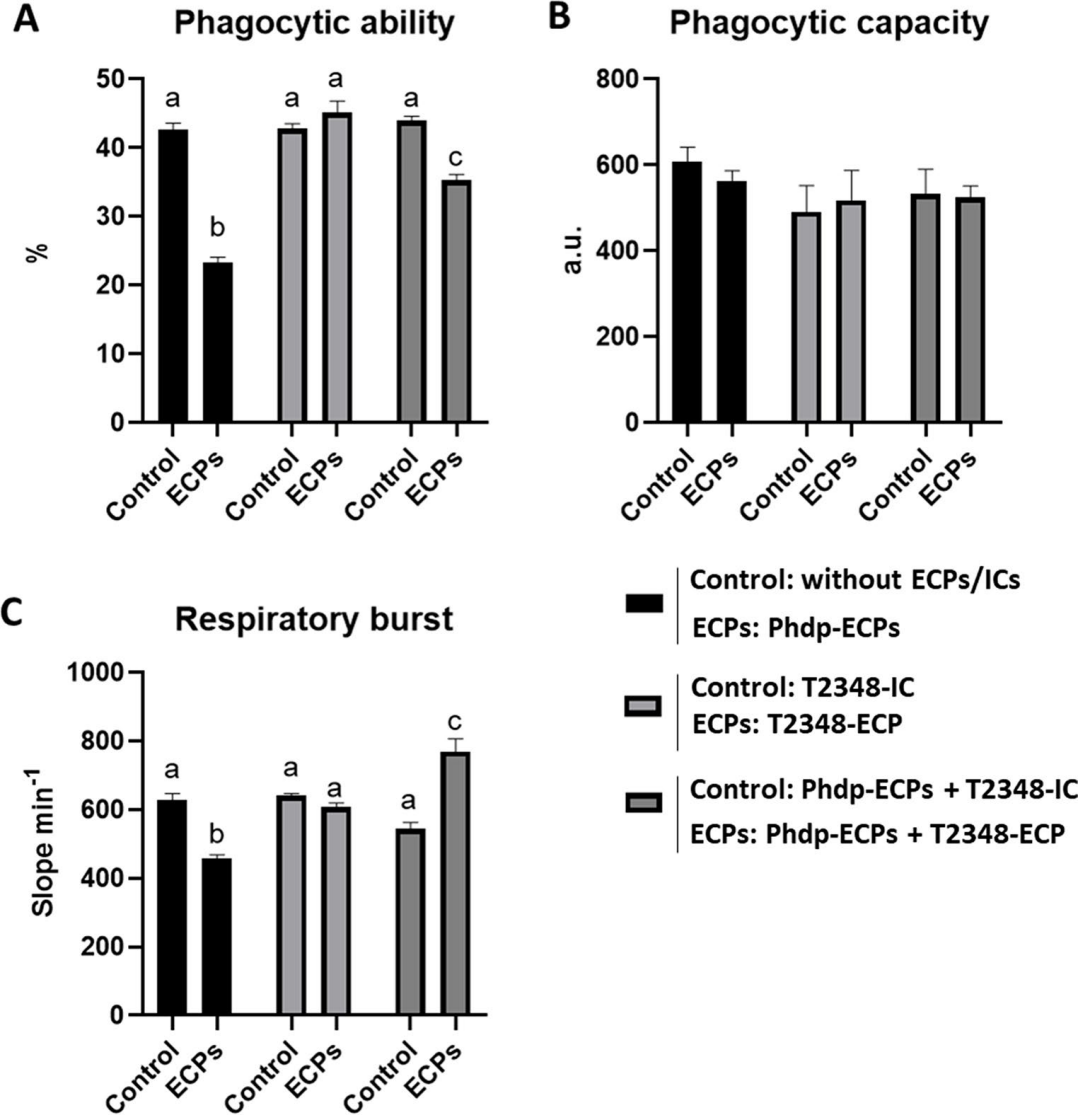
Cellular innate immunity parameters of European sea bass (*Dicentrarchus labrax*) head kidney leucocytes (HKLs) incubated for 24 h with different ECPs and ICs. Bars from left to right indicate: Control (HKLs) and ECPs (HKLs + Phdp-ECPs); Control (HKLs + T2348-IC) and ECPs (HKLs + T2348-ECP); Control (HKLs + Phdp-ECPs + T2348-IC) and ECPs (HKLs + Phdp-ECPs + T2348-ECP). **A**. Phagocytic ability, **B**. Phagocytic capacity and **C**. Respiratory burst. Results are expressed as means and error bars in the columns indicate the standard error of the means (*n* = 6). Different letters indicate significant differences (*p* < 0.05) between groups (Control and ECPs)

The percentage of phagocytosis and respiratory burst of HKLs are summarized in Fig. 6. Phagocytic ability decreased significantly when HKLs were incubated with Phdp supernatant (Phdp-ECPs) (Figs. 6A and 7A). However, no significant differences were observed between HKLs incubated with FM1548-ECP or T2348-ECP compared to control HKL cells (Figs. 6A and 7A). In contrast, phagocytic ability was significantly increased in HKLs incubated with Phdp supernatant combined with FM1548-ECP (Phdp-ECPs + FM1548-ECP) or T2348-ECP (Phdp-ECPs + T2348-ECP) (Figs. 6A and 7A), regarding the effects observed in HKLs exposed to Phdp supernatant (Phdp-ECPs). ICs did not affect phagocytic ability in any case (Figs. 6A and 7A). As for phagocytic capacity, the same trends as just described for phagocytic capacity were observed, but in none of the cases significant differences were obtained (Figs. 6B and 7B).

Finally, the effects on the respiratory burst (Figs. 6C and 7C) were very similar to those described for phagocytic ability. The respiratory burst of HKLs significantly decreased when incubated with Phdp supernatant (Phdp-ECPs). However, no significant differences in this parameter were observed between HKLs incubated with FM1548-ECP and T2348-ECP, neither their ICs (Figs. 6C and 7C). In contrast, respiratory burst was significantly increased in HKLs incubated with Phdp supernatant combine with FM1548-ECP (Phdp-ECPs + FM1548-ECP) or T2348-ECP (Phdp-ECPs + T2348-ECP) (Figs. 6C and 7C).

### Relative Gene Expression of Aip56 Gene

**Fig. 8:**
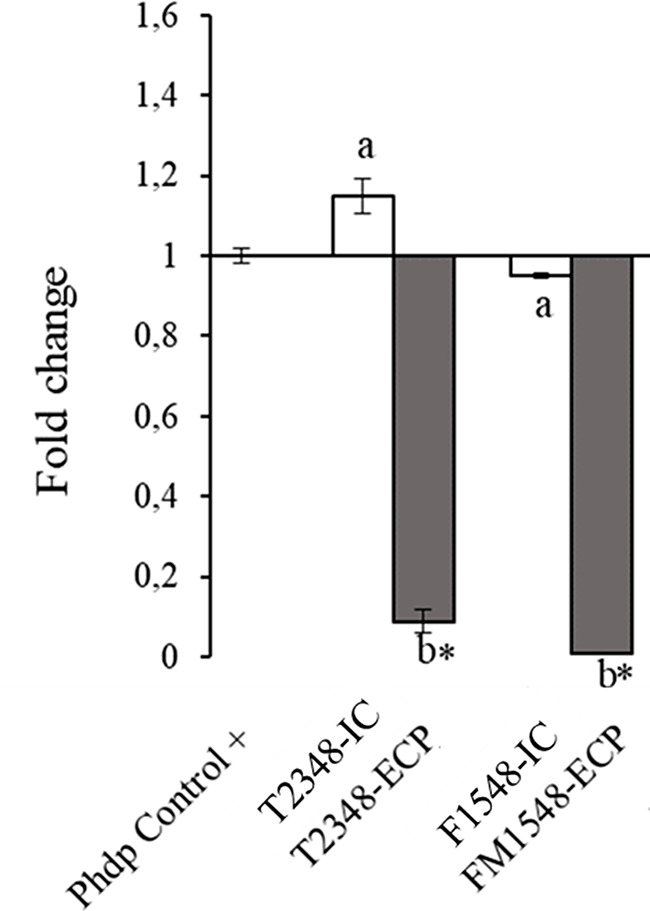
Relative expression of Phdp *aip56* gene after adding SpPdp11-ECPs or their ICs (µg protein µL-1) extracted from *S. putrefaciens* Pdp11 probiotic strain cultured under different conditions. “Phdp Control +” indicates expression value without ICs or ECPs added (control). The expression values ​​for the ICs and the SpPdp11-ECPs are represented with white and grey bars, respectively, and indicate the different culture conditions assayed (T2348 and FM1548). Letters in the nomenclature indicate the culture medium: T: TSAs and FM: mix of aquafeed and a blend of microalgae added medium, and numbers stand for temperature (ºC) and incubation time (h). Letters (a, b) indicate significant differences (one-way ANOVA; *p* < 0.05) between SpPdp11-ECPs and their respective ICs. Asterisk (*) indicates significant differences (one-way ANOVA; *p* < 0.05) from control (Phdp Control +). Values ​​represent the mean ± standard error of the mean (SEM) of three independent experiments with five technical replicates (*n* = 5)

Effects of all SpPdp11-ECPs and their ICs tested on the relative transcription of the *aip56* gene encoding the Phdp AIP56 toxin are shown in Fig. 8. While ICs (T2348-IC and FM1548-IC) did not affect the expression of the *aip56* gene, with regard to ECP treatments, both FM1548-ECP and T2348-ECP, showed a highest ability to significantly reduce *aip56* gene transcription.

## Discussion

Postbiotics have been shown in studies to have antibacterial (pathogenic and spoiler bacteria) effects, preventing and controlling infectious diseases [42]. The influence of culture conditions on the production of postbiotics is widely reported [27, 28], suggesting that the activity, quantity and type of these derived products are mainly related to the type of bacterial strain and type of culture medium. Therefore, our research group have studied the in vitro capabilities of SpPdp11-ECPs obtained from the probiotic *S. putrefaciens* Pdp11 grown under different cultivation conditions. Some of these capabilities were hydrolytic, antagonistic, antiviral or non-cytotoxic effect, as well as the inhibition of pathogen biofilm formation or quorum-quenching (QQ) capacity [27, 28, 43, 44]. These previous results support the SpPdp11-ECP conditions, T2348-ECP and FM1548-ECP, selected for the present study and their possible implications against Phdp virulence. Phdp infections are characterized by the occurrence of generalized bacteriemia and extensive cytopathology with abundant tissue necrosis and pathological changes mainly attributed to AIP56 toxin [16], which has a significant apoptotic activity against fish macrophages and neutrophils, especially in sea bass specimens [16].

Firstly, the in vitro MIC assays showed absence of antagonistic effect against Phdp cultures in both SpPdp11-ECP samples, T2348-ECP and FM1548-ECP, obtained from the probiotic *S. putrefaciens* Pdp11. These results contrast with those obtained by Chabrillón et al. [17], who reported an antibacterial effect of SpPdp11 cells when in contact with cells from two Phdp strains. Thus, the results obtained could suggest that the anti-Phdp molecules contained in the SpPdp11-ECPs studied could be highly diluted or that this antagonistic effect was due to a contact-dependent growth inhibition (CDI) system, a mechanism described in many Gram-negative bacterial species to compete with other cells [45–47]. In this case, effector toxins can be delivered directly to neighbouring bacterial cells as a result of direct physical contact with them [48, 49]. However, future research will be necessary to verify these mechanisms of action in SpPdp11.

Then, FM1548-ECP and T2348-ECP were selected for application in ex vivo assays with sea bass head kidney leucocytes (HKLs) challenged with Phdp supernatant (Phdp-ECPs), since the AIP56 toxin is an exotoxin, abundantly secreted by a type II secretion system [50] as an extracellular protein [51, 52]. Since the cytotoxic effect of any product should be tested before considering it as a potential candidate for any clinical or biological application [53], the results obtained showed that, in any case, SpPdp11-ECPs, T2348-ECP and FM1548-ECP, were toxic to HKLs. On the contrary, HKLs that interacted with Phdp supernatant (Phdp-ECPs) showed considerable increased percentages of dead cells, specifically by apoptotic mechanisms (around 60%). This destruction of HKLs can be related with the AIP56 toxin, and its apoptosis induced mechanism that leads to massive phagocyte lysis [54] and consequently to depletion of the phagocytic response, unrestricted multiplication of the pathogen and exposure of host tissues to cytotoxic molecules released by lysing phagocytes [16]. This effect is reduced when SpPdp11-ECPs, T2348-ECP and FM1548-ECP, were added to HKLs separately or combined with Phdp supernatant (Phdp-ECPs + T2348-ECP and Phdp-ECPs + FM1548-ECP). Thus, the results obtained could suggest a specific interaction in which SpPdp11-ECPs (T2348-ECP and FM1548-ECP) mitigate the Phdp virulence. These results could be related by transcriptional regulations of the encoded aip56 gene [55, 56] or subsequent steps such as hydrolysis or structural C- or N- terminal modification of the secreted AIP56 exotoxin that avoid its internalization [16] among others possible mechanisms of inactivation, which need to be further investigations.

Furthermore, the percentage of death cells is similar with those of apoptotic cells after 24 h incubation of HKLs in presence of SpPdp11-ECPs (T2348-ECP and FM1548-ECP), as well as in presence of Phdp-supernatant (Phdp-ECPs). In addition, the fact that the percentage of necrotic cells was low after HKLs incubation with the different ECPs, T2348-ECP and FM1548-ECP, suggests a programmed cell death related with apoptosis. These results agree with previous ones obtained by do Vale et al. [54] who reported the destruction of leucocytes, macrophages and neutrophils, of sea bass by apoptosis. This apoptosis is triggered by the AIP56 exotoxin, what suggests a possible implication on this term. However, it has been reported that the toxin triggers apoptosis of host leucocytes (macrophages and neutrophils) through a process that, in vivo, culminates with secondary necrosis of the apoptotic cells contributing to the necrotic lesions observed in the diseased animals [57]. Secondary necrosis is an autolytic process where cells disintegrate and release their components. This process takes place when no scavengers are involved, and the entire apoptotic program has been executed [58]. Flow cytometry assays may suggest that the toxin is capable of producing the apoptosis of HKLs after 24 h incubation, but not to discriminate the phase of secondary necrosis. It would be interesting to perform other types of experiments and analysis that would allow us to deduce if the toxin is also capable of finally producing the secondary necrosis of the apoptotic HKLs.

In terms of cell-mediated immunity, phagocytosis and subsequently HKL respiratory burst were significantly reduced by Phdp supernatant (Phdp-ECPs). During HKLs incubation with Phdp supernatant (Phdp-ECPs), the apoptotic effect from the AIP56 exotoxin could lead to the cleavage of NK κβ p-65 [57], inducing the activation of different caspases, leading to the collapse of the mitochondrial membrane potential and high production of reactive oxygen species (ROS) [59]. However, HKL respiratory burst significantly decrease when they were incubated with Phdp supernatant (Phdp-ECPs), which could be explain with the presence of described enzymes that reduce ROS levels in virulent Phdp strains [14]. On the other hand, phagocytosis and respiratory burst of HKLs was not influenced by SpPdp11-ECPs (T2348-ECP and FM1548-ECP), while combined with the Phdp supernatant (Phdp-ECPs + T2348-ECP and Phdp-ECPs + FM1548-ECP), significantly increased both, phagocytosis and respiratory burst of HKLs compared to those incubated with Phdp supernatant alone. In this sense, the role of the SpPdp11-ECPs tested is relevant in cell-mediated immunity. Normally, phagocytic leukocytes increase its oxygen consumption after pathogen-associated molecular patterns (PAMPs) activation through NADPH-oxidase induction for producing different ROS during phagocytosis [60]. Simultaneously, functional respiratory burst responses are usually correlated with the activation of signalling pathways, including the release of inflammatory cytokines, which can ameliorate phagocytes mobilization in fish [61]. Thus, T2348-ECP and specially FM1548-ECP could enhance or accelerate the cell-mediated immune response of HKLs during infection, since the respiratory burst response is observed when combined SpPdp11-ECPs with Phdp supernatant.

According to the current results obtained, the SpPdp11-ECPs assayed, T2348-ECP and FM1548-ECP, suggest a reduction in the AIP56 exotoxin effect. For this, these SpPdp11-ECPs were then evaluated on the Phdp relative transcription of *aip56* gene. Thus, both FM1548-ECP and T2348-ECP, exerted a similar and a greatest significant reduction of *aip56* gene transcription. Thus, the mechanism responsible for this down-regulation is unknown, new studies should be developed at the biochemical level in order to understand the mechanisms involved in the regulation of its expression. However, the current results coincide with those previous that demonstrated the ability of SpPdp11 to resist challenges with Phdp [9, 22]. Owned-studies demonstrated how SpPdp11 interfere with the adhesion of Phdp to mucous surfaces of farmed fish such as Senegaelse sole and gilthead seabream [23]. The challenges carried out by our group have been administered the probiotic SpPdp11 in the diet [20, 22, 62, 63], an appropriate route of administration for their ECPs in further in vivo studies. Apart from this, when aquafeed is added to the medium with a blend of microalgae (FM medium), gene transcription down-regulation was slightly greater than when T medium. Anyway, the positive effects of the SpPdp11-ECPs from the FM medium (FM1548-ECP) pave the way in relation to fish feeding. The replacement of fishmeal or fish oil by other natural dietary supplements include microalgae take more attention in the field for promoting aquaculture sustainability and fish health benefits [64].

In conclusion, previous results by our research group demonstrated the postbiotic potential of *S. putrefaciens* Pdp11 is affected by culture conditions. Thus, two different postbiotics, as SpPdp11-ECPs, FM1548-ECP and T2348-ECP, were selected for the present study. The postbiotic potential of these SpPdp11-ECPs has demonstrated a mitigation of dead and apoptotic cells in sea bass leucocytes, as well as an increase in the phagocytosis and the respiratory burst in these cells. This ability has been correlated with an interference with the transcription of the gene encoding the AIP56 exotoxin, a highly relevant virulence factor of Phdp. These findings lead to new studies for applying SpPdp11-ECPs as postbiotics on the treatment or prevention of fish diseases, considering that a future in vivo test will be proposed to corroborate the protective effect of ECPs against Phdp infection on cultured fish, providing key findings to promote and improve future research.

## Electronic Supplementary Material

Below is the link to the electronic supplementary material.

Supplementary Material 1

## Data Availability

No datasets were generated or analysed during the current study.
