# Supplementary material for: An ex vivo Approach in European Seabass Leucocytes Supports the in vitro Regulation by Postbiotics of Aip56 Gene Expression of Photobacterium damselae subsp. piscicida
Source: Probiotics Antimicrob Proteins. 2024 Apr 23;17(4):2097–111. doi: 10.1007/s12602-024-10255-x (PMC12405407; doi:10.1007/s12602-024-10255-x)
Supplement: Supplementary file 1 — Supplementary Material 1 [file 12602_2024_10255_MOESM1_ESM.docx]

**Jorunal research:** Probiotics and Antimicrobial proteins

**An *ex vivo* approach in European seabass leucocytes supports the *in vitro* regulation by postbiotics of aip56 gene expression of *Photobacterium damselae* subsp*. piscicida***

Marta Domínguez-Maqueda^a*^, Cristóbal Espinosa-Ruíz^b^, María Ángeles Esteban^b^, Francisco Javier Alarcón^c^, Silvana T. Tapia-Paniagua^a^, María Carmen Balebona^a^, Miguel Ángel Moriñigo^a^

^a^Departamento de Microbiología, Facultad de Ciencias, Instituto Andaluz de Biotecnología y Desarrollo Azul (IBYDA), Universidad de Málaga, Ceimar-Universidad de Málaga, Málaga, Spain; [martadm@uma.es](mailto:martadm@uma.es), [stapia@uma.es](mailto:stapia@uma.es), [balebona@uma.es](mailto:balebona@uma.es), [morinigo@uma.es](mailto:morinigo@uma.es).

^b^Departamento de Biología Celular e Histología, Facultad de Biología, Universidad de Murcia, Murcia, Spain; [cer48658@um.es](mailto:cer48658@um.es), [aesteban@um.es](mailto:aesteban@um.es).

^c^Departamento de Biología y Geología, Universidad de Almería, Ceimar-Universidad de Almería, Almería, Spain; [falarcon@ual.es](mailto:falarcon@ual.es).

***Corresponding author:** Silvana Teresa Tapia Paniagua

**Email address:** stapia@uma.es

**Postal address:** Departamento de Microbiología, Facultad de Ciencias, Instituto Andaluz de Biotecnología y Desarrollo Azul (IBYDA), Universidad de Málaga, Ceimar-Universidad de Málaga, 29071 Málaga, Spain

**Table S1.** Ingredient composition of the experimental diet used for the aquafeed culture media of ECP samples extraction. Aquafeed provided by Tecnovit Lifebioencapsulation

| **Composition** | **Content (%)** |
| --- | --- |
| Fish meal LT94 | 10,00 |
| Soycomil R | 15,00 |
| Wheat gluten | 17,00 |
| Pea protein concentrate | 5,00 |
| Soy meal (50%) | 20,00 |
| Wheat meal | 14,14 |
| Fish oil | 7,00 |
| Soy oil | 4,50 |
| Colza soil | 4,50 |
| Vitamins and minerals | 1,00 |
| Vitamin C | 0,05 |
| Vitamin E | 0,01 |
| Methionine | 0,50 |
| Monocalcium phosphate | 1,30 |
| Microalgaes |  |
| Summarized | 100,00 |
